# Extracellular Vesicles and Circulating Tumour Cells - complementary liquid biopsies or standalone concepts?

**DOI:** 10.7150/thno.73400

**Published:** 2022-08-01

**Authors:** Artur Słomka, Bingduo Wang, Tudor Mocan, Adelina Horhat, Arnulf G. Willms, Ingo G.H. Schmidt-Wolf, Christian P. Strassburg, Maria A. Gonzalez-Carmona, Veronika Lukacs-Kornek, Miroslaw T. Kornek

**Affiliations:** 1 Department of Pathophysiology, Nicolaus Copernicus University in Toruń, Ludwik Rydygier Collegium Medicum in Bydgoszcz, 85-067 Bydgoszcz, Poland.; 2 Department of Internal Medicine I, University Hospital Bonn of the Rheinische Friedrich-Wilhelms-University, 53127 Bonn, Germany.; 3 Institute of Molecular Medicine & Experimental Immunology, University Hospital Bonn of the Rheinische Friedrich-Wilhelms-University, 53127 Bonn, Germany.; 4 Octavian Fodor Institute for Gastroenterology and Hepatology, Iuliu Haţieganu, University of Medicine and Pharmacy, 400162 Cluj-Napoca, Romania.; 5 Department of General, Visceral and Vascular Surgery, German Armed Forces Hospital Hamburg, 22049 Hamburg, Germany.; 6 Department of Integrated Oncology, Center for Integrated Oncology (CIO), University Hospital Bonn of the Rheinische Friedrich-Wilhelms-University, 53127 Bonn, Germany.

**Keywords:** CTC, extracellular vesicles, exosomes, microvesicles, ectosomes, biomarker, liquid biopsy, personalized medicine

## Abstract

Liquid biopsies do promise a lot, but are they keeping it? In the past decade, additional novel biomarkers qualified to be called like that, of which, some took necessary hurdles resulting in FDA approval and clinical use. Some others are since a while around, well known and were once regarded to be a game changer in cancer diagnosis or cancer screening. But, during their clinical use limitations were observed from statistical significance and questions raised regarding their robustness, that eventually led to be dropped from associated clinical guidelines for certain applications including cancer diagnosis. The purpose of this review isn't to give a broad overview of all current liquid biopsy as biomarkers, weight them and promise a brighter future in cancer prevention, but rather to take a deeper look on two of those who do qualify to be called liquid biopsies now or then. These two are probably of greatest interest conceptually and methodically, and likely have the highest chances to be in clinical use soon, with a portfolio extension over their original conceptual usage. We aim to dig deeper beyond cancer diagnosis or cancer screening. Actually, we aim to review in depth extracellular vesicles (EVs) and compare with circulating tumour cells (CTCs). The latter methodology is partially FDA approved and in clinical use. We will lay out similarities as taking advantage of surface antigens on EVs and CTCs in case of characterization and quantification. But drawing readers' attention to downstream application based on capture/isolation methodology and simply on their overall nature, here apparently being living material eventually recoverable as CTCs are vs. dead material with transient effects on recipient cell as in case of EVs. All this we try to bring in perspective, compare and conclude towards which future direction we are aiming for, or should aim for. Do we announce a winner between CTCs vs EVs? No, but we provide good reasons to intensify research on them.

## Introduction

The history of liquid biopsies in cancer is funded on a historical misunderstanding through many decades how the term liquid biopsies is actually defined vs. how it has been used nowadays as outlined by Todd M. Morgan in his review article from 2018 and by others. Dr. Morgan made his point that defining “what constitutes a liquid biopsy is important here.” 1. Furthermore, he wrote, that “[t]he term biopsy implies direct measurement of a tumo[u]r, so the liquid biopsy marker should be restricted to tests with specificity approaching that of a tissue biopsy”. In the light of this definition, it's practically impossible to use the term liquid biopsies in association with extracellular vesicles (EVs) if going in line with Dr. Morgan. Probably, it should be substituted with the term personalized cancer diagnostics, or as Dr. Morgan suggested, “…the term liquid biopsy is becoming as commonplace as precision medicine, ”that's because it probably is.”^1^.

Despite this interesting opinion, majority of experts and their countless expert reviews including our own, original research titles and other peer-reviewed publications are still using and accepting the term liquid biopsy to define different kind of biomarkers that are obtained from patients applying non-invasive (urine sample, smear) or minimal-invasive (puncture to draw blood) procedures and which are associated with patients' health condition, present, past or prognostic 2.

One of the oldest reports on liquid biopsies was published 1966 by Wichelhausen RN *et al*., which is matching the widely used definition and isn't a biopsy in the light that patient's cell tissue was obtained and then eventually further cultured and finally analysed accordingly 3. From far bigger ramification was a publication from 1990 in which Partin A.W. and colleagues reported the quantitative assessment of prostate specific antigen (PSA) in serum samples in association with prostate cancer tumour volume and differentiation and as benign prostatic hyperplasia volume 4. Interestingly, at that time the authors reported discrepancies between pathological stage and serum PSA that might be explained by a decrease in production of PSA with increasing histological grade according to authors of this study. Few decades later, 1991, the study results were repeated on a broader scale and published in *New England Journal of Medicine*, a highly respected and very influential clinical peer-review journal, hence, eventually becoming the gold standard in prostate cancer screening *via* a liquid biopsy 5. Going in line, 1994, US Food and Drug Administration (FDA) approved PSA in conjunction with a digital rectal exam (DRE) to test asymptomatic men for prostate cancer 6. PSA is potentially one of the first liquid biopsy marker as approved by the FDA. And probably a story of success saving countless of men lives.

Another liquid biopsy cancer biomarker that was once regarded to be the one that might bring screening and diagnosis in case of hepatocellular carcinoma (HCC) to another level was Alpha-fetoprotein (AFP). This biomarker was even recommended to be included to several international and national guidelines as provided by various organizations as the American Association for the Study of Liver Disease (AASLD) and the European Association for the Study of the Liver (EASL) besides others 7-9 at that time. Due to the promising results, the 2003 HCC guidelines from the British Society of Gastroenterology recommended both this biomarker and abdominal ultrasound for HCC diagnosis 10. During the course of using this biomarker, several concerns regarding sensitivity and specificity and usefulness of cut-off values appeared, so that finally this marker was soon questioned and eventually dropped for HCC screening and wasn't recommended anymore until today by AASLD or EASL 11-13. Very unfortunately, AFP had been shown to be relatively insensitive as it is only elevated in 40-60% of HCC cases, thus HCC patients exhibit normal AFP serum levels, particularly during early stage disease 14, 15. Additionally, AFP levels can also be found elevated in non-HCC patients, including non-cancerous chronic liver diseases, intrahepatic cholangiocarcinoma and metastatic colon cancer 16. In sum clinical practice guidelines do not recommend AFP (or any other biomarker for that matter) for the diagnosis of HCC 12.

Besides these two proteins based liquid biopsy cancer biomarkers, some other methodical interesting liquid biopsy biomarker in cancer were actually studied, being somehow non-invasively taken as cervical smear that is routinely assessed and used to explore feasibility of experimental use of those samples for DNA testing using both Digene Hybrid Capture assay (DHCA) and polymerase chain reaction (PCR) techniques at that time, 2001, to detect high-risk human papillomaviruses (HPVs) DNA 17. From nowadays perspective probably to be regarded as another milestone in liquid biopsy in cancer.

Especially in the last 1 ½ decades, liquid biopsy biomarker in cancer took another spin, another boost by taking advantage of novel tracers of various tumours that might be potentially successfully utilized. The “tumour circulome” comprises circulating tumour cells (CTCs), extracellular vesicles (EVs), cell-free tumor DNA (ctDNA), cell-free DNA (cfDNA), circulating tumour RNA (ctRNA), and tumour-educated platelets (TEPs) 18. All of those biomarkers are in agreement kind of liquid biopsies and as Dr. Morgan would say, precision medicine. However, in this review we would like to discuss differences and similarities between EVs and CTCs. We note comprehensively the limitations and advantages of two upcoming liquid biopsies in cancer screening and diagnosis. Both are somehow ancestors of the tumour mass itself, directly, being a tumour cell as CTCs are or indirectly, as being derived from a tumour cell as EVs are. One of the main differences between both markers is from numeric nature. In the following paragraphs two of those, EVs and CTCs, will be introduced in depth since both are sharing some interesting common features regarding their analysis.

## EVs - a star is born

54 years ago, 1967, Wolf P described something like as 'platelet dust' as a trivial by-product of cell degradation in his preparations 19. His electron microscopic data revealed lipid‐rich particles that may originate from the osmophilic granules of platelets and interestingly the “liberation of this material” as he called, is the result of 'activation' 19. Nowadays, we call them extracellular vesicles (EVs) and typically activation is one of the major mechanisms *in vivo* and *in vitro* besides others to induce the release of small EVs (sEVs) or large EVs (lEVs) 20. 2011 we published that the induction of lEVs-release by various mechanisms as donor cell activation, as induction of apoptosis in donor cells might eventually result in lEVs differing in the capability to induce fibrolysis in recipient cells *in vitro* 21. At that time, we called those EVs 'microparticles' and others 'microvesicles' or 'ectosomes'. Today's International Society For Extracellular Vesicles (ISEV) guidelines, published by ISEV members as us, with the purpose to frame an urgent needed standardisation, actually agreed on to call them correctly as lEVs 22.

During the last decade EV research intensified by a lot, EV research caught finally attention of many research communities including liver research communities and others 23. Thus, advanced methodologies enabled to categorize EVs into sEVs, typically consistent of exosomes, and smallest microvesicles (MV). On the contrary, lEVs are consistent of microvesicles aka microparticles (MPs)/ectosomes 20, 24, 25. This careful differentiation implies already that distinction between sEVs and lEVs, isn't razor sharp and exactly defined by a size number. Some might say 100nm might be the border, other 150nm 22, 26. However, exosomes and MPs/MVs can be distinguished clearly by their biogenesis. MPs/MVs could range in sizes from around 100 to 1000nm and are shed directly from the cell membrane by a “budding process” during cellular activation or early apoptosis 20. Exosomes are the smallest vesicles, usually below 100 nm, and formed in endosomes and are stored within multi-vesicular bodies (MVB) that release their contents into intracellular space upon fusion with the cell membrane 27. Several markers for exosomes have been described including head shock protein HSP70 and the integrins as CD63, CD9, CD8 28-30. However, some of these might be expressed on MVs/MPs as CD9 and CD81 31.

Apart from EV size and EV marker issues that are in detail unresolved, lEVs are somewhat representing its donor cell with its membrane-associated proteins on a smaller scale, making lEVs into an appealing field of research. sEVs and lEVs contain lipids, cytosolic proteins, some messenger RNAs and microRNAs 20, 22, 32. Once EVs were considered to be a kind of a cellular waste system, removing unneeded cytosolic proteins, some messenger RNAs and microRNAs. This idea is still under discussion 33. Apparently, EVs are more than that. EVs are orchestrating many physiological or pathological effects by their cargo and membrane composition and are a novel horizontal cell to cell communication route, including for the communication between tumour cell and tumour niche, inducing tumour tolerance. Apart from that, eventually its worth to highlight their potential for early cancer screening, cancer diagnosis, especially before metastasis takes place 34. Until now, not a single human body cell was reported to be incapacitated of the ability to release EVs including tumour cells, “underlining the attractiveness of these vesicles to use as novel minimal invasive biomarkers not only in liver tumours but also in non-malignant liver diseases” 25. In other words, EVs might be an outstanding tool as an integral part of a new generation of liquid biopsy cancer biomarkers. Moreover, newest work done by von Felden *et al.* on exosomal unannotated small RNA clusters associated with circulating extracellular vesicles detect early stage liver cancer has given us a preview how beautifully future biomarker studies will take advantage of artificial intelligence (AI) to find novel profiles that helps to manage cancer patients 35. It is foreseeable that AI might be a game changer 36. However, validation cohorts/studies are still needed to verify AI driven data bank analyses. Additionally, it is noteworthy to hint that some evidence are given that exosomal proteo-transcriptome may vary compared to their donor cell as shown for 12 cancer cell lines and in human prostate cancer, which is unexpected. How much relevance it could have, e.g implicate a given limitation to which extend EVs are mirroring their donor cells, remains open 37*.*

The pathophysiological role of EVs is probably manifold and in depth discussed elsewhere. Many *in vitro* and *in vivo* animal experiments were done demonstrating a possible role of EVs in extracellular communication between cells proximal or distal, downstream pathway activation in EV recipient cells or even contributing to tumour tolerance 30, 34. But, those reports could only give a glimpse that EVs, lEVs or sEVs, have actually a more pronounce pathophysiological role then thought and from far bigger clinical interest beyond cancer screening. One of the most prominent examples of EVs capability acting as a novel treatment was shown exemplarily 2017. Kordelas L. *et al.* performed a first human interventional study where mesenchymal stem cell derived EVs (MSC-EVs) were administered in case of steroid refractory graft-versus-host disease (GvHD) 38. How precisely MSC EVs did act was debatable, some thought that MSC EVs might be rather the source of a substance that was released by MSC. However, MSCs were isolated from bone marrow donors, expanded and conditioned supernatants were collected being potentially MSC-EV rich. A dose, 1 unit of MSC EVs was defined as the amount of MSC EVs released by 4 × 10^7^ for patient's body weight. This highly cited publication demonstrated a successful experimental clinical application of MSC-EVs where MSC-EVs decreased probably indirectly numbers of patient-derived peripheral blood cells, which secreted pro-inflammatory cytokines IL-1β, TNFα and IFNγ, likely contributing in modulating patient's immune status that led to significant improvement of clinical GvHD symptoms 38. Following this report, it is fair to conclude that EVs, here MSC derived EVs might play an important future role as a novel therapy option, e.g. in personalized medicine.

## CTCs, established and accepted concept

Circulating tumour cells (CTCs), commonly a subset of malignant cells, that are typically disconnected from their primary tumour and circulating in the peripheral blood, can be phenotyped according their surface antigen status or analyzed at their expression-, protein- and genomic level allowing their assessment of their dynamic status of the respective patients at different cancer stages and cancer heterogeneity 39, 40. Noteworthy, CTCs were first time described by Ashworth T.J. in 1869 in blood of a metastatic cancer patient indicating their role in cancer spread and by doing so paving the ground for modern CTC based diagnostics 41. Thus, rarely, CTCs may also appear in premalignant conditions, under conditions where “potential” CTCs were detected without any molecular proof of malignancy at this stage 42. Nowadays, CTCs are regarded to be one of the most prominent kinds of liquid biopsy and their enumeration offers a simple, highly standardized and vigorous way to do precise medical disease pre-diagnosis, prognosis, and therapy response assessment 39, 43, 44. Several applications in various tumour entities had been published so far. Some of those are very compelling and some are showing interesting conceptual extensions of the common CTC diagnostic routine. The authors covering certain aspects of how CTCs could be potentially applied in pre-diagnosis, prognosis, and therapy response assessment have summarized those.

Considering the blood micro-environment in which CTC circulating, it is quite challenging to find a viable CTC maker to eliminate interference of billions of white blood cells, non-malignant endothelial cells, stem cells, or other blood cells as erythrocytes, thrombocytes, leucocytes etc. These CTC markers should be at best located on the CTC cell membrane, therefore easily accessible and robust enough expressed on those CTCs without pathological inhibition. In other words, its shouldn't be present on non-malignant cells, for example, epithelial markers which could be absent on mesenchymal leukocytes but presented on cancer cells or *vice versa*, depending if CTC will be positive or negative selected 45. Newer CTC capturing strategies are either based on their physical properties as size (ISET: isolation by size of epithelial tumour cells) 46, as elasticity and deformability 47 and others that aren't depending on their surface tumour specific antigen profile. Therefore CTC isolation can be generally divided into label and label-free detection as reviewed in detail by Habli Z. *et al.* 47. Independent of such methodical advantages, the most established and most robust CTC capture methodology is probably the immunobead assay using epithelial cell adhesion molecules (EpCAM) to select EpCAM^+^ CTCs (positive selection) and simultaneously to exclude CD45^+^ white blood cells (negative selection) 48. Keeping in mind that 1995 Gross H.J. *et al.* demonstrated that utilizing flow cytometry could in fact discriminate rare cancer cells from other cells in blood and bone marrow introducing the usage of CD45 and “multiple markers, each identified by a separate color of immunofluorescence (yellow and two shades of red) tri-fluorochromes” 49; at that time a remarkable achievement in flow cytometry. Approximately one decade later, finally, in 2004 the CellSearch^TM^ system was introduced and first data in Clinical Cancer Research published 48. The CellSearch^TM^ system, as an only Food and Drug Administration (FDA)-cleared immunobead assay detecting EpCAM^+^CK^+^CD45^-^ CTCs has been designed to enumerate the number of CTCs in 7.5ml of whole blood under the assumption that under non-pathological conditions EpCAM^+^CK^+^CD45^-^ cells will be not present in the peripheral blood. The sensitivity exceeds 90% in metastatic breast, in metastatic prostate, and in metastatic colorectal carcinoma studies 48. Currently, the CellSearch^TM^ is still regarded as the gold standard by many experts in the field. Typically, in brief, CTCs will be captured, enriched, and fluorescently stained by the automated autoprep system, and last enumerated by the semi-automatic CellSpotter Analyzer 48, 50.

To overcome shortcomings of EpCAM as used by CellSearch^TM^ system and others, the detection error of isolation by size of epithelial tumour cells (ISET) due to size overlapping between CTCs (12 -35µm) and some monocytes /eosinophils (12-20µm) 46, a lot of attention was shifted to the modern nanoelectromechanical CTC chip (NELMEC) and believed that NELMEC can solve aforementioned problems because compare with tumour cells, lower membrane capacitance and higher cytoplasmic conductivity in leukocytes would give less observable movement in the impedance of the SiNG electrode 51. Another chip based solution, CTC iChip, actually combined size-based enrichment with either EpCAM positive enrichment or CD45 negative depletion with a reported yield of rare cells of 97% and processing rate of 8ml of whole blood/h 52.

To date, the inclusion of CTCs in the clinical assessments for the management of colorectal and breast cancers has not been accepted by the American Society of Clinical Oncology (ASCO) Tumor Marker Guidelines Committee. In 2007, CTCs and disseminated tumour cells (DTCs) were just cited in ASCOS's recommendations on cancer markers 53. On the contrary, lately, the American Joint Committee of Cancer (AJCC) proposed a new category for TNM staging in breast cancer (M0(i+) as defined by CTCs or DTCs, if evidences of distant metastases are missing 54. Likewise, no other committees recommended CTC to diagnose cancer or make therapy decisions, but still possible all phases of CTC cancer guidelines will be formulated following the advantage of mature CTC techniques in the near future 55. The reasons might be manifold, the limited possibility to detect cancer at an early timepoint under the caveat of cancer screening? Some speculated that the heterogeneity of the clinical studies regarding patients/CTC donor selection might play a role asking for CTC guideline (CTC Guide) on study design and study report 55.

Some authors try to extend the common CTC diagnostic routine to additional cancer entities as non-small cell lung cancer (NSCLC) 56, 57, hepatocellular carcinoma (HCC) 58, 59, pancreatic ductal adenocarcinoma (PDAC) 60, 61 besides other cancer entities. Most of these studies were carried out using peripheral blood as a CTC source, in NSCLC pulmonary venous PV-CTC counts were assessed being not significantly related to the personal age, gender, smoking status, even pathological stage, an increased PV-CTC count would be closely associated with the higher risk of cancer recurrence 62. NSCLC patients with a PV-CTC count higher than 7/7.5ml blood volume post R0-resection are designated to be more frequent postoperative medical examination, due to a higher risk for NSCLC recurrence 62. Furthermore, few studies could demonstrate the benefit of CTC in pre-screening efforts providing evidence that the existence of CTC was actually detected by an average of 3.2 years earlier than lung nodules discovered in the subsequent CT scan in COPD patients. The authors named this CTC as sentinel CTCs 63.

## CTCs vs EVs, a direct comparism

### Advantages, limitations pros and cons

Though CTCs and EVs are very different in many ways, living material, ongoing metabolism and reproducible, proliferative vs. non-living material, kind of smallest traces of cells, besides obviously being different in size, macro vs nano-sized. Nevertheless both, CTCs and EVs, share some methodical similarities how to be identified, some minor conceptional and mythological differences how to be phenotyped. Incommon, both are taking advantage of utilizing surface antigens as available on their membrane surface 64. The widely used CellSearch^TM^ is designed using surface antigens such as EpCAM or CK and others as discussed as a kind of positive selection and CD45 for negative selection of leucocytes, besides DAPI, an intracellular nucleus staining as discussed before 48. This nucleus staining capability of CTCs is in EVs obviously not given, since EVs are lacking a nucleus and larger genomic material except minor genomic fragments 65, 66. Additionally, and probably one of the major differences is that an intracellular staining in case of EVs, likely to be called an intravesicular staining, wasn't reported so far to our knowledge, since an intracellular staining relies generally speaking “to poke holes” into the cell membrane using mild membrane solubilizers as Tween 20, as Saponin, as Digitonin and as Leucoperm (0.5% v/v in PBS) 67. However, a very recent publication demonstrated that EVs bound to a coverslip overnight at 4 degrees Celsius were fixed with paraformaldehyde and were permeabilized using 0.1% Saponin for downstream imaging purposes 68. Without fixation, such detergence will eventually destroy as reported for sodium dodecyl sulfate (SDS), an anionic detergent, the structural integrity of nano-sized EVs and random those to be useless for quantification efforts 69.

Typically, both type of liquid biomarker, CTCs and EVs, had been phenotyped by flow cytometry by several researchers around the globe using various available models of flow cytometers including advanced imaging flow cytometry as with various resolutions regarding lower detectable size boarder 70-73. A publication in Nature 2015 on GP1^+^ (Glypican) sEVs, exosomes, was eventually the most recognized publication and a kind of kick off in EV based cancer diagnosis 74. On the contrary, 2004 can be regarded as the year when CTCs had their breakthrough by recognition and approval by the FDA. Nevertheless, the usage of sEVs in prostate cancer diagnosis received FDA acknowledged by granting Breakthrough Device Designation to Bio-Techne ExoDx Prostate IntelliScore (EPI) test, 48, 75. Nevertheless, CTCs had been so far researched for a longer time period than EVs as a kind of cancer liquid biopsy biomarker in patients' blood. Hence it is not surprising that the methodology of CTC isolation and phenotyping is more advanced and to a higher degree standardized. Including FDA approved for the detection of several metastatic cancer entities in the USA. In contrast, the EV research field is somehow still exploring and elaborating the usefulness of EVs in cancer diagnosis and especially with a strong emphasis on prognosis 44. EVs are still somewhat experimental and guidelines were given to the EV field by the International Society for Extracellular Vesicles (ISEV) 2014 and 2018 76, 77. Of note CTC methodology is not final set, neither in case of EVs. New combinations of CTC antigens have been investigated allowing diagnosis and prognosis in case of additional cancer entities, yet not FDA approved 72.

Hypothetically, there is actually an overlap given of used antigens on CTCs and EVs and being specific for cancer entities. The classical CellSearch^TM^ pan cancer marker is EpCAM, reliably used for the detection of CTCs in co-junction with CD45 and DAPI and others (experimental) 44, 48, 58. Interestingly, EpCAM on lEVs was insufficient in discrimination between investigated cancer entities as colorectal-, as non-small cell lung-, as pancreas carcinoma and thyroid nodules, kind of abnormal growth of thyroid cells forming a lump within the thyroid gland, typically non-malignant 78. In fact this was foreseeable and the need of combinations of antigens was discussed 2014 already 79. Only combination of antigens being simultaneously present on the same large EV, here AnnexinV, CD147 (Emmprin) and EpCAM were sufficient to separate patients suffering from thyroid gland and cancers 78. Additionally, other combinations were reported being specific to a certain extend for biliary malignancies, including the EV based differentiation of HCC from cholangiocarcinoma (CCA) including intrahepatic CCA 73, 80. Applying cancer entity specific antigens on CTCs was meanwhile achieved by taking advantage of hepatocyte markers as Glypican3 and ASGPR1, in combination with EpCAM utilizing flow cytometry for experimental purpose 72, 81. ASGPR1 in combination with EpCAM was also successfully applied to detect HCC derived lEVs 80.

Some researchers didn't rely on initial EpCAM-based capture of CTCs allowing them to identify additional CTC populations being associated with cancer progression. Armstrong A.J. *et al*. explored not only CTCs in patients with metastatic CRPC co-expressing EpCAM or CK, but rather E-cadherin, mesenchymal proteins as Vimentin, as N-cadherin and O-cadherin, thus CD133 as well 82. All of them eventually missed by the FDA- approved CellSearch^TM^ methodology and other on CTC isolation and numeration depending methods heavily relying on EpCAM. Amstrong's *et al.* data suggests that CTCs from common epithelial malignancies co-express epithelial and mesenchymal markers, suggesting that EMT/MET transition is likely contributing to metastatic progression. From importance is, that EpCAM is lost earlier than cytokeratin during the epithelial-to-mesenchymal transition resulting in escape from EpCAM-based CTC capture 83. To our best knowledge EMT/MET transitions surface antigens on EVs were not explored yet.

Another flow cytometric advantage and applicable on CTCs, eventually isn't yet reproduced on EVs, the so-called discrimination of high expressing and low expressing CTCs 84, up to date not achieved on EVs. Scientists around the world can easily providing ample of examples where it does matter if a cell's antigen is high or low/dim expressing 85, 86. No reports are available if CellSearch^TM^ is capable to do so would be off-label usage. An experimental setting in which the blood waste was enriched with cancer cell line cells (blood waste discarded by CellSearch^TM^) was collected and passed through a micro-sieves and accounted. In fact, the CellSearch^TM^ cartridge effectively recovered EpCAM^high^ tumour cells, whereas the EpCAM^low^ cells are mainly recovered by micro-sieve 87. This might be from relevance, since EU-FP7 CTC-Trap program suggested that CTCs expressing high levels of epithelial cell adhesion molecule, EpCAM^high^ compared to EpCAM^low^ CTCs were associated with a different clinical outcome as given in median survival in metastatic prostate and breast cancer patients. In favor of EpCAM^high^ CTCs, those were strongly related to shorter survival 84.

As shown, EpCAM does play a prominent role in the FDA approved CellSeach^TM^ system. The hypothetical question that must appear, is obviously, if the CellSearch^TM^ System and other immunoaffinity - positive selection/enrichment based systems will evolve further into a system that is more capable to detect specific cancer entities by utilizing another selection of antigens present on cancer derived CTCs, for example Glypican-3, ASGPR1 and HepPar1 as for liver cancer derived CTCs 88-90. Of note such move has successfully to pass clinical phases prior FDA approval and clinical use. Would such evaluation of the CellSearch^TM^ system give physicians an advantage in liver cancer diagnosis and prognosis? It is debatable and financial interest might play a role.

However, as discussed above both, CTCs and EVs, can be used for cancer diagnostic applications and of note cancer prognosis 44-46, 62, 74, 80. Furthermore, at this juncture, we have a real field to maneuver, as many studies have indicated that both structures with high sensitivity and specificity can be used as a liquid biopsy in the diagnosis and prognosis of patients' outcomes and responses to treatment 36, 44, 91. Nevertheless, many meta-analyses have confirmed the unique diagnostic and prognostic value of CTCs 92-95. The same properties are attributed by meta-analyses to circulating EVs 96-99. We conjecture that both of these laboratory markers will soon be introduced into run-of-the-mill clinical diagnostics and that the interpretation of the results may be helped by artificial intelligence and machine learning 30. In case of cancer prognosis, there might be another prognostic advantage in case of CTCs. CTC clustering. Interestingly, many recent studies have shown that at the beginning of metastasis, CTC clusters are more dangerous than single cells 100. Recently, it has been identified that Na^+^/K^+^ ATPase inhibitors can dissociate CTC clusters into individual cells, thereby inhibiting the spread of cancer, as this might affect DNA hypermethylation at the binding sites of OCT4, NANOG, SOX2, SIN3A and, so on 101. There's lots of evidence that the formation of tight clusters means shorter remission time, which can use for treatment efficacy monitoring. For example, a patient with lung cancer produced tighter CTC clusters in culture after accepting several cycles of nivolumab, and later PET and CT examinations also further confirmed that the patient had multiple metastases 102. Obviously, those discoveries provide direction for different therapeutic areas. Again, CTC analysis permits cluster analysis if cell clusters weren't excluded by the used flow cytometry gating strategy. On EV level, nano-size level, we cannot exclude or include reliably EV clusters, typically EVs are ranging a size from 100-1000nm in case of large EVs, giving ample or room for EV duplets or triples. An optical EV flow cytometer as provided by AMNIS^TM^ and optimized for the detection of EVs 70, could potentially allow the identification of EV doublets or EV clusters, still doubting on their naturally existence or rather being an artefact due to the isolation process? No experimental data is available on that matter.

Another interesting aspect is, that the source of CTCs and EVs, not on a cellular level, rather, if isolated from peripheral blood (PB), from cancer draining venous blood (DVB) or other organ specific fluids (bile, cyst liquid) might make a difference regarding their diagnostic performance. It was concluded that CTCs numbers may differ after transition through organs, especially high vascularized organs as liver and lung as shown for human colon carcinoma cell line, KM12-HX s, after intraportal vein (i.p.v.) or intravenous (i.v.) injection into rats 103. Some researchers might consider that peripheral blood might be easier assessable, but numbers of CTCs in cancer draining venous blood (DVB) could evolve to a more robust diagnostic and prognostic performance. Hence, Tsutsuyama M. *et al*. compared CRC derived CTCs isolated either from DVB or from PB, and demonstrated higher CTC numbers in DCB. Furthermore CTCs numbers in DVB the level of CTCs continued to rise with the cancer deterioration 104. Noteworthy and somehow a limitation is the fact, that in this experimental setting, DCB was obtained from the mesenteric vein immediately after tumour resection 104. Going in line in it was reported that higher CTC counts in portal venous blood collected during pancreaticoduodenectomy in periampullary or pancreatic adenocarcinoma without metastases as could predict liver metastases post-surgery 105. Similar results were reported a year earlier in patients resectable pancreatic cancer 106. The question that arises now is obviously if EV numbers will be higher in DVB compared to PB as reported for CTCs? Hypothetically the answer should be probably. Surprisingly, in resectable non-small cell lung cancer (NSCLC) difference in size between sEVs isolated from tumour-draining pulmonary vein blood compared to PB were observed. Actually tumour-draining pulmonary vein blood was enriched with sEVs (30-50nm) and smaller EV size was linked to tumour relapsed and shorter overall survival 107. Unfortunately, the authors didn't discuss the cause of such size differences. 2017 another interesting observation was reported and highly published despite its methodical simplicity, which might let to reasonable faster transfer from an experimental stage towards clinical use. sEV concentrations were compared as detectable in bile and PB in malignant biliary stenosis as pancreatic cancer as cholangiocarcinoma. As expected, sEV concentration in bile was significantly higher and could distinguish malignant vs. non-malignant CBD stenosis with 100% accuracy 108.

The above-discussed differences between CTCs and EVs might be considered minor to a certain degree. Other differences as described next, might be considered major, since CTCs and EVs might obviously not being capable to be used as the other one and *vice versa*. One of the major differences is, that EVs might be used as a drug delivery system. On the contrary CTCs might be not a desired candidate for drug delivery after any thinkable *ex vivo* manipulation for obvious reasons. Paclitaxel-mediated mesenchymal stromal cells possible exert an excellent anti-tumour effect by obtaining drugs and subsequently packaging them in EVs 109. Interestingly, it was suggested in order to overcome possible cross-species adverse EV tolerance effect the usage of bovine milk-derived sEVs for drug delivery 110. This might be an alternative to artificial produced EVs consuming needed resources as lipids which are in need for mRNA based vaccines 111.

Additionally, EVs might own certain capabilities to support organ/tissue damage repair due to the used EV origin in case of mesenchymal stromal/stem cell (MSC)-derived sEVs as reviewed by Varderidou-Minasian S and Lorenowicz M.J. 112. Just to highlight the capabilities of MSC-EVs the group around Giebel B. successfully administered MSC-EVs in case of steroid refractory graft-versus-host disease (GvHD) 38. Until this it was believed that MSC might be a therapy option in severe therapy-refractory acute GvHD. However, how precisely MSC did act was debatable, some thought that MSC might be rather the source of a substance that was released by MSC. However, MSCs were isolated from bone marrow donors, expanded and conditioned supernatants were collected being potentially MSC-EV rich. A dose, 1 unit of MSC-EVs was defined as the amount of MSC-EVs released by 4×10^7^ for patient's body weight. This highly cited publication demonstrated a successful experimental clinical application of MSC-EVs where MSC-EVs decreased numbers of patient-derived peripheral blood cells, which secreted the pro-inflammatory cytokines IL-1β, TNFα and IFNγ, likely contributing modulating patient's immune status and significant improvement of clinical GvHD symptoms 38.

Obviously, CTCs cannot be used to be *ex vivo* manipulated and then administered back to the donor patient hoping for a kind of cancer cure. But circulating MSC if robust phenotyped, could be likely isolated with a CellSearch^TM^ like methodology too, thus recovered from the CellSearch^TM^ cartridge and *ex vivo* manipulated or simply serving as MSC-EVs donors and stored for later, similar to the collection of umbilical cord haematopoietic stem cells (HSCs). Many research groups around the globe are working downstream solution on various recovery strategies of CTCs from the cartridge or utilizing other devices, commonly aiming for a possible expansion of CTCs *in vitro* due to the low numbers of rare CTCs in cancer patient's blood as discussed by Sharma S. *et al*. 113. Typically followed by several applications as drug testing, as expression arrays, as genomic analysis for prognosis or as estimating possible drug effectives on designated cancer entities 114-117. Of note expression arrays and genomic analysis can be done with single cells technology since CTCs are rare and low in numbers. How rare? Recently, in a German study metastatic prostate cancer patients were associated with a median CTC count of 4 (range, 0-820), and a mean CTC count of 27 as baseline before treatment 118. Similarly, low numbers were associated with prostate cancer 119, However, the initial CellSeach^TM^ publication from 2004 reported for metastatic carcinoma entities as prostate, breast, lung, colorectal, ovarian and others a mean of 60 ± 693 CTCs per 7.5 mL and a used cut-off of 2 CTCs per 7.5mL 48.

Of note, expression analysis on mRNA and protein level, miRNA arrays including small noncoding RNA 32, 120, 121 can be obviously applied to EVs too as shown by many scientists. Aiming for the detection of cancers 74, cancer entity discrimination 73, 80 or for prognostic proposes 98, 122, 123. Of note, the question is, how feasible EV expression or genomic arrays are in term of a future clinical application? Simplicity might be desired here, as the detection of biliary cancer on the base of total EVs in bile or overall costs per sample 108.

All these possible applications pointing towards personalized medicine and will be discussed in depth later. However, drug testing can be done only on living material as CTCs, since reverse engineer of a living cell by adding up EVs should be regarded as impossible. Therefore, drug testing *ex vivo*/*in vitro*, to choose the most effective anti-cancer drug or it's concentration (e.g. for individualized drug susceptibility test) will be only achieved with CTCs 114, 124-126. *In vivo*, the effectives of given treatment/drugs, in terms of therapy monitoring can be achieved with CTCs 127 and EVs 128.

Besides additional surface antigens that are actively explored being suitable for CTC enrichment, certain soluble proteins, as cytokines in conjunction with CTCs have been investigated as well, potentially associated with cancer progression and metastasis. 2016 the SUCCESS Study group reported that in pre-therapy primary breast cancer soluble IL-1α was associated with CTC presence in peripheral blood but not within the lymphatic-system indicating IL-1α might suit as a marker for lymphatic cancer invasion. CTC-positive patient no lymph node involvement were reported with high levels of IL-1α, whereas those with lymph node involvement expressed low levels of IL-1α 129. The SUCCES study group members concluded that reduced levels of IL-1α might be a crucial factor to generate a tissue microenvironment that stimulates cancer expansion 129. Besides IL-1α, IL-17A was linked with disease-free survival in patients with colorectal carcinoma (CRC), potentially serving as a surrogated marker in combination with CTC in CRC. Same group reported that ablation of IL-17A combined with rGM-CSF effectively suppressed CTCs and prevented organ metastasis in a CRC mice model indicating an novel CRC treatment option too 130.

On the contrary, in PDAC Glyp^+^ sEVs were correlating with CA19-9: However, Glyp^+^ sEVs alone were associated with a sensitivity and specificity of 100% 74. Another synergistic example between a liquid biopsy marker, here lEVs and a soluble antigen was reported recently. Soluble AFP could enhance the discrimination between HCC and CCA in case of AnnV^+^CD44v6^+^ lEVs and AnnV^+^CD44v6^+^CD133^+^ lEVs, thus no correlation between AFP values and the investigated EV subpopulation were observed 73. For a diagnostic application in real life it's doubtful if such synergistic effects are desired, since the initial liquid biomarker as CTCs, as EVs, have to be measured plus an additional liquid biomarker as soluble a protein, as a cytokine. A perfect, a robust liquid biomarker alone should provide the needed information if the investigated specimen is harboring a tumour, underlying tumour entity and mirror tumour dynamics, growth/shrink.

**Figure 1:**
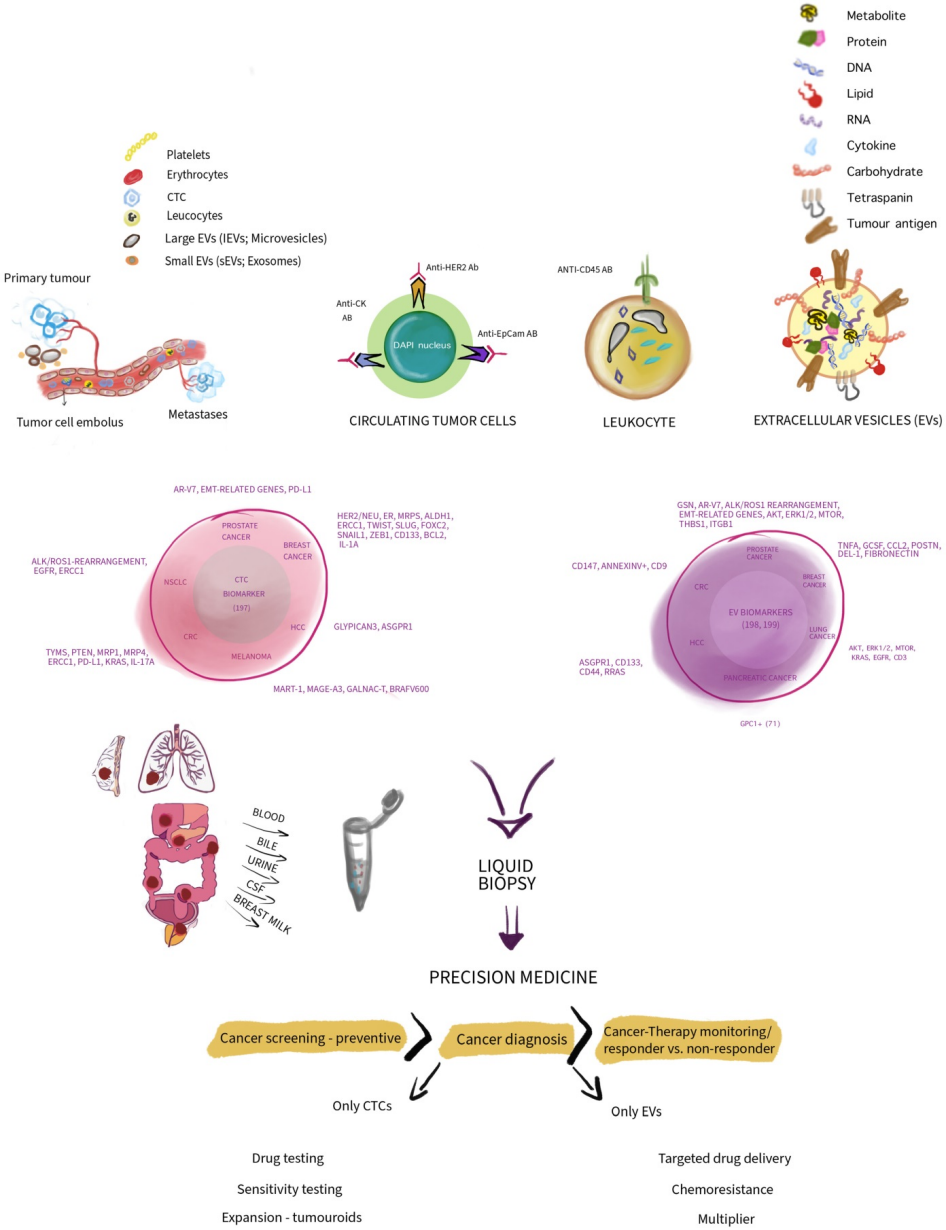
A simplified concept depicting circulating tumour cells (CTCs) and extracellular vesicles (EVs) in a comparative manner highlighting and summarizing their differences in composition. Thus, presenting some markers that are in use to associate CTCs and EVs with various cancer entities. Additionally, indicating how CTCs and EVs as biomarkers may be utilized in the context of precision medicine in cancer and associated downstream application. Note, depicted sizes do not show size and diameter differences in reality *in vivo*. We thank Wioleta Chomko for transformation our scientific thoughts into art.

Several thoughts are summarized and depicted in Figure 1.

### Aiming for realizing personalized medicine with CTCs

CTCs are not only restricted to cancer screening and cancer diagnosis/prognosis based on phenotyping surface antigens on those CTCs which various methods and devices as discussed above in detail. Moreover, CTCs are harboring genomic DNA, miRNA, mRNA, various kind of proteins and lipids 39. At the end they are human living material with an ongoing metabolism. Whereas in EVs genomic DNA is generally absent as cell organelles as mitochondria too, except for DNA fragments due to volume restriction 39. In agreement EVs are death material without an ongoing metabolism. Best case, ATP that is included in EVs might be used up for proteins ensuring temporary an asymmetric membrane composition regarding phosphatidylserine (PS) which is an anionic phospholipid found in cell membranes and EVs 24, 131, 132. Nevertheless, these characteristics of both biomarkers is allowing several downstream applications after their successful isolation or enrichment. However, we have to keep in mind that CTCs are restricted to an overall cancer phenotype allowing disconnection, dislocation and migration though surrounding tissue infiltrating blood vessels 133. Such restriction wasn't reported for EVs. EVs were released in colorectal carcinoma (CRC) patients suffering from a non-metastatic or metastatic CRC 78. Such tumour cell behavior is typical for metastasis, hence metastatic cancer entities.

CTC isolation and recovery are currently in focus and ongoing testing is in progress permitting several downstream applications beyond sequencing genomic material as harbored in CTCs or expression arrays on protein or mRNA level including miRNA profiling. At best, this kind of downstream investigations can be done with little CTC material with low CTC numbers or even as single cell analysis 134. Providing information regarding various characteristics that individual CTC might have and limiting therapy success if resistance to a chosen drug might be given. Certain mutations might give input regarding chemoresistance, invasion, EMT, extravasation, evasion and migration for instance as reviewed by others in depth. Such information is truly part of personalized medicine effort 135.

Why are CTC numbers crucial? This is due to the fact that CTCs are very rare. Even if 7.5mL of whole blood is the recommended volume for CTC detection by the FDA approved CellSearch^TM^ system, the reported results with this commercially available system is typically ranging from 1-100 and only few specimens are actually acceding >100 CTCs/7.5mL whole blood 48, 119. A multi centric study reported astonishing low number of CTCs (<6 CTCs/7.5mL) in the majority of cancer patients (78%) suffering from various cancers as bladder, breast, prostate, pancreatic, adenocarcinoma, colorectal, ovarian, lung, head and neck and others 136. 2015 Miyamoto D.T. *et al.* reported a successful approach based on the recovery of 77 CTCs out of 13 prostate patients (6/patient) and subsequently expression analysis on mRNA level may help to predict resistance of anti-androgen therapy in spite of patients' heterogeneity regarding androgen receptor-dependent and -independent alterations 137. On the contrary EVs are actually a multiplier, any cell, including tumour cell may release several sEVs and lEVs, likely increasing the chance to isolate and detect these nano-sized tracers of their donor cell. Typically, detected EV numbers are in a range of 10^8^ to 10^12^ per mL serum or plasma 26, 138, of note depending on the investigated EV populations and subpopulations. Additionally, we must account that CTCs half-live is limited, somewhat restricted to 1-2.4 hrs as observed in patients suffering from breast cancer 139. Whereas tumour derived EVs may be detectable up to several days after a R0 resection suggesting a half live of days 74, 78, 80.

In spite of CTC's numbers, CTC are living material that could be expanded in a certain time frame depending on their initial numbers 140. Apparently, low initial CTCS numbers have to be traded for higher expansion phase and higher CTCs numbers fortunately for a shorter expansion phase of those. Current research is primary focusing on novel clinically suitable methods ensuring higher initial CTC numbers, but secondary and from equal importance to shorten CTC's expansion phase, permitting sufficient CTC numbers to test simultaneously and in a short period of time several drugs, granting that the best drug will be administered to the patient 141. Probably, such an approach would completely be depending on a successful expansion phase allowing a comprehensive drug sensitivity profile. Such effort could significantly reduce time and rule any non-responders to the selected anti-tumoural therapy 127. Such decision-making of the probably most effective drug is of course very much desired in case of rapid progressing cancers as small cell lung cancer (SCLC) and others. Of note antigen selection for CTC enrichment is crucial. Recently, Lee H.-L. *et al.* enriched EpCAM^+^CD45^-^ SCLC CTCs for a further downstream application as a spheroid proliferation assay. Noteworthy, spheroid proliferation was established within 4 weeks and associated with the expression of TTF-1, synaptophysin and PDL-1, permitting further assessment of drug sensitivity of cisplatin and etoposide 115. CTCs were enriched using a bead-based CTC enrichment strategy based on the RosetteSep^TM^ CTC Enrichment Cocktail kit. Therapy monitoring identifying drug responders from non-responders is desired and doable and part of a personalized medicine effort. 2014 Barbazán J. *et al*. published a study for which 50 mCRC patients were recruited and receiving a typical first systemic chemotherapy, typically fluoropyrimidines (fluorouracil or capecitabine) alone or in combination with oxaliplatin/irinotecan and biological targeted therapies (bevacizumab, cetuximab) 127.

But what about the feasibility of large-scale testing of FDA approved drugs on patient's own tumour cells? Is it doable? Probably yes, such libraries are in use 142. Several CTC derived tumour cell lines were established. Eventually, Cayrefourcq L. *et al.* reported first establishing a cell line from CTCs of a colon cancer patient 143. As we know, each tumour is somewhat highly personalized, carrying an individual mutation profile besides needed mutations that are typically for malignant cells. Does mutation profiling of pre-established tumour cell lines be beneficial. Rather not, but probably giving direction. Recently, a new concept emerged, creating tumour mass *in vitro* faster, due to the fact that the initial tumour cell numbers are much advantageous. Classical needle biopsies or resected tumour tissue may lead to sufficient numbers for seeding and growing of so called organoids 144, here tumouroids, on which drugs may be tested prior use in patient's since patients' own biopsy made it possible 145, 146. This concept is surely and reasonably an alternative, if the trade of needle biopsy for liquid biopsy will result in a shorter expansion phase. Gaining time for the sake of cancer patients is key. However, that might be a desired goal, proof of concept was done with tumour tissue that was obtained during an R0 tumour resection ensuring high amounts of tumour cells for drug testing. Such an approach resulted in a total of 1500 tumouroids that were used for drug screening demonstrating that in this highly personalized approach a combined treatment with AKT and mTOR inhibitors may be a promising strategy for the treatment of patient's CRC 147.

Different cancer-driving mechanisms impact differently on the genomic rearrangements and the genetic heterogeneities of CTCs, that's the rationale behind aiming to maximize diagnostic precision. The high reliable genome integrity index (point mutations, gene amplifications, and whole genomes of single cells) was defined by different molecular assays to assess the molecular heterogeneity of single CTCs from metastatic cancer patients to suggest the evolution of personalized medication 148. In line, a recent study showed that the up-regulation of Notch activity and the increase of uPAR^+^/int-β1^+^ CTCs are newly discovered CTC molecular signals in breast cancer patients who had brain metastasis, which can be used as a magic tool for early detection of micro-metastases in brain setting, or used to make a reasonable therapeutic decision and supervise the response during drugs management 149.

Taken together expansion of CTCs may permit comprehensive molecular profiling of CTCs. Comprehensive in that way, that not few cells or one CTC cell was analyzed, but a certain number which will result in more robust data. However, we have to keep in mind that expansion of CTCs *in vitro* as organoids, precisely tumouroids may acquire additional mutations/gene due to the length of expansion phase. Broutier L. *et al*. reported that in case of primary liver cancer (PLC) on average approx. 92% of variants as seen in patient's tissue were retained in the corresponding early tumouroid cultures (< 2 months), and > 80% after months of additional expansion 150. However, important PLC antigens as typical HCC markers as AFP and as GPC3 and hepatocyte markers as ALB, as TTR, as APOA1 and as APOE were highly expressed in those HCC tumouroids and ductal markers as expected down regulated. Broutier L. *et al*. concluded that “PLC-derived organoid culture system faithfully recapitulates and maintains the transcriptomic alterations present in the individual patient's tumour subtype.”^150^.

Nevertheless, certain CTCs have a magic that is surrounding them, but CTCs number are low in spite of newest efforts to increase those numbers *via* sub-sequential expansion of CTCs. But what might bring the future? Could be once that we simply go to dialysis center not because of a kidney problem, but rather to get rid of CTCs? To prevent metastatic seed in patients at risk due to a metastatic cancer phenotype? At the same time those CTCs could be recovered and obviously since we wash the whole blood volume for CTCs, captured CTC numbers could be expected to be much higher likely permitting downstream applications as discussed. A time costly expansion of those CTCs could be avoided. This idea of so-called CTCs hemodialysis isn't new. Wang X. *et al.* discussed their hypothesis 2013 in greater detail 151. However, 2021 Jarvas G. *et al*. published a modification of hemodialysis membranes for such purpose to capture EpCAM^+^ CTCs in an experimental setting. They used HCT116 cancer cells both into buffer solution and added to whole blood. One endpoint was flow cytometry to assess efficiency. Efficiency was given as 69% and approx. 2.1 × 10^6^ cells/m^2^ as absolute cell capture capacity potential that would result in, if assuming an average patient with 80 kg body and approx. 5 L of blood and a threshold of 5 CTCs/7.5 mL blood, nearly in approx. 3000 CTCs 152, which is a clear improvement. But is it superior than organoids/tumouroids or rather a convenient starting point increasing the chances for a successful organoids/tumouroids culture? Of note if we nail it down to patients' view on choosing between a convenient CTCs hemodialysis or an invasive needle biopsy or tumour resection in order to harvest sufficient primary tumour cells for an organoid/tumouroid culture, we might have a winner here.

In contrast to CTCs, EVs are conveniently present in all body fluids 153 and a multiplier of the donor-cells as discussed above. Hence, numbers are sufficient in contrast to CTCs. However, EVs must be considered to be somewhat experimental and clear guidelines and gold standards must be set including on EV storage. Since thawing and freezing cycles are affecting EV quality, numbers and hence reproducibility as reviewed by others in depth 154, 155. In the next two paragraphs we would like to discuss potential EV applications as in case of chemoresistance and targeted drug delivery. Two aspects in which CTCs aren't a match, 1^st^ due to low numbers and unresolved expansion limitations and 2^nd^, obviously, CTCs aren't a vector candidate that could be given back to the CTC donor patients.

### EVs and chemoresistance

Contemporary oncology offers a cancer patient several treatment options, of which chemotherapy is stillthe gold standard 156. Substantive restriction in clinical use of cytostatic drugs is resistance of neoplastic cells to their effects, which is often manifested by resistance to cytostatic drugs with multifarious structures and divergent mechanisms of action 157-159. This phenomenon is called multiple drug resistance (MDR), and for a long time, researchers have tried to elucidate the complex molecular mechanisms underlying cancer cell resistance to chemotherapeutic agents 158, 159. Over the last decade, considerable attention has been paid to the role of EVs in MDR 160, 161. Although EVs are a relatively heterogeneous population, and various EV forms can be investigated in the context of chemoresistance, it is appropriate to clarify that the preponderance of the experimental studies described how sEVsand their associated non-coding RNAs (ncRNAs) regulate chemoresistance. Therefore, in this part of our narrative review, we present succinctly how cancer cells acquire resistance to chemotherapeutic agents through the active transmission of molecular information by sEVs.

Undeniably, sEVs are diligent players in developing chemoresistance in cancer cells 162, 163. Nonetheless, some conditions must be met for sEVs, here precisely exosomes, to modulate this phenomenon. First, after being formed in the cell within multivesicular bodies (MVBs), the relevant molecule determining chemoresistance must be inserted into the sEV structure, after which exosomes are expelled from the cell into the extracellular environment 164, 165. Second, sEVs must be absorbed by another cell, where the carried molecules undergo various processes, depending on their nature, that the cell phenotype changes 164, 165. Typically, sEVs that determine chemoresistance are released predominantly by cancer cells with a natural resistance to specific anti-cancer drugs. Nevertheless, the components of the tumour microenvironment (TME) can also, as shown for sEVs, condition resistance to cytostatics 166-169. Such properties are attributed to, inter alia, carcinoma-associated fibroblasts (CAFs) 166, 167, tumour-associated macrophages (TAMs) 168, and mesenchymal stem cells (MSCs) 169. Nevertheless, sEVs released by tumour cells that fulfill a cardinal role in disseminating the chemotherapeutic resistance, and although these changes require sophisticated cytomolecular machinery, the phenomenon appears to be prevalent. As the cutting-edge research shows, the tumours that can zealously generate EVs with such attributes is practically limited to the types of human cells undergoing malignancy 170, 171. The ability to release in particular sEVs with chemoresistance-modulating properties has been described mostly in the case of gastrointestinal (GI) cancers, including tongue squamous carcinoma (TSC) 172, esophageal cancer 173, 174, gastric cancer (GC) 175, 176, colorectal cancer (CRC) 177, 178, hepatocellular carcinoma (HCC) 179, 180, and pancreatic cancer (PC) 181. Exosome-mediated chemoresistance has also been reported in cancer cells of ovarian 182 and cervical origin 183, as well as in non-small cell lung cancer (NSCLC) 184, 185 and glioma 186. This phenomenon can also occur in the course of breast cancer (BC) 187 and acute myeloid leukemia (AML) 188.

However, the rudimentary question is, how do exosomes superintend the chemoresistance? The literature analysis indicates that two main mechanisms are involved; however, the studies discussed the first one more. This cardinal mechanism involves various forms of ncRNAs, which are transported through exosomes to sensitive cells, changing their properties 173, 178. Therefore, it must be considered here that exosomes constitute a guided missile aimed at chemotherapeutic-sensitive cells. Exosomal microRNAs (miRs) confer chemoresistance via inhibiting cancer suppressor genes in recipient cells 173, 178. For example, exosomal miR-193 silences transcription factor AP-2 gamma (TFAP2C) in the cisplatin (CDDP)-sensitive esophageal cancer cell line, thus removing CDDP's inhibition of the cell cycle and increasing chemoresistance 173. In turn, CRC cells acquire resistance to oxaliplatin (OX) by suppressing an apoptosis-related gene, programmed cell death protein 10 (PDCD10) *via* exosomal miR-46146 178. A growing body of evidence reveals that exosomal long ncRNAs (lncRNAs) and circular RNAs (circRNAs) vehemently manipulate the genetic material of cancer cells to achieve chemoresistance 189, 190. Mechanistically, both types of exosomal ncRNAs, through miR sponging (acting as a competing endogenous RNA; ceRNA), lead to increased expression of oncogenes 183, 184. An example is the excellent work by Luo X. *et al.*, which suggested that exosomal lncRNA HNF1A antisense RNA 1 (lncRNA HNF1A-AS1) promotes CDDP resistance in cervical cancer (CC) cells by sponging miR-34b to promote the expression of tuftelin1 (TUFT1), which exerts oncogenic roles 183. Successively, exosomal hsa_circ_0014235 constitutes the ceRNA for miR-520a-5p, thus increasing the expression of cyclin-dependent kinase 4 (CDK4) in NSCL, leading to resistance to CDDP 184. Phenotypically, such actions of exosomal ncRNAs lead to increased cell proliferation, migration, and invasion, as well as inhibition of cancer cell apoptosis 183, 184. Other examples of chemotherapeutic agents against which cancer cells become resistant through exosomal ncRNAs are temozolomide (TMZ) 186, 5-fluorouracil (5-FU) 191, and gemcitabine (GEM) 181. Interestingly, in breast cancer, exosomal lncRNA small nucleolar RNA host gene 14 (SNHG14) mediates resistance to trastuzumab, the monoclonal antibody used in its treatment 192. The second mechanism is the promotion of chemoresistance through exosomal cargo proteins 171. Undoubtedly, the mechanisms of action of individual proteins are highly diverse. For example, exosomal fibroblast growth factor 2 (FGF2) from bone marrow stromal cells (BMSCs) is taken up by leukemia cells, which results in their resistance to tyrosine kinase inhibitors (TKIs) 193. Exosomes can also transport phosphorylated signal transducer and activator of transcription 3 (p-STAT3), increasing the CRC resistance to 5-FU 194.

### EV in targeted drug delivery

Advantageously, two can play at that game! If, as proven in the preceding subsection, sEVs may significantly modulate tumour cell response to cytostatics, why not use them as drugs themselves or as drug haulers to cancer cells? Theoretically, the idea is not new as various drugs have long been incorporated into liposomes—a synthetic spherical vesicle 195, 196. Nevertheless, sEVs and EVs in general might be regarded as universal transmitters of information between cells, and this property should be unquestionably exploited in the study of tumours. In all probability, in cancer therapy, our knowledge that naturally formed sEVs expressing ncRNAs can increase the sensitivity of cancer cells to cytostatics may constitute basis of search for their application in clinical oncology. The second approach is to incorporate cytostatic drug molecules into the structures of EVs. These are multistage processes that require extensive researcher experience and high-class laboratory apparatus. Notwithstanding these constraints, many studies are already registered in *ClinicalTrials.gov* by researchers who wish to keep abreast of the new developments in this field, although a significant part of these studies still pertains to the role of EVs in the diagnosis and prognosis of neoplastic diseases. In addition, several excellent reviews on the potential use of EVs as cancer treatment options have been published 197-200. These manuscripts presented advanced data on the engineering of EVs as drug carriers and their potential clinical application in various disorders. In our narrative review, we described, following the structure of the prior subsection, which cytostatic drugs, regardless of the research model, can be delivered to cancer cells by EVs, and the results that are obtained from such transport. The technical details of preparing or modifying EVs are deliberately omitted here as these issues are fully described and seem to be discussed more often than the therapeutic options themselves and the potential effects at the molecular or cellular level.

A review of experimental studies plainly shows that the exosomal transport of two cytotoxic drugs is the most frequently described, namely doxorubicin (DOX) belonging to anthracyclines 201-205 and paclitaxel (PTX) belonging to the taxane family of anti-neoplastic agents 205-209. Other cytotoxic drugs such as 5-FU 210 or a representative of antimetabolites (e.g., gemcitabine) 211 are, in this context, much less tested. There are two overarching goals behind the research conducted in this area. Firstly, a cytostatic drug attached to exosomes reaches cancer cells more effortlessly, showing greater than standard cytotoxicity and possibly higher clinical effectiveness 201, 203, 205, 210, 211. Secondly, this form of drug transport may substantially reduce the side effects of cytostatics in cancer patients, especially cardiotoxicity of doxorubicin, likely due to preventing leakage of the drug before it reaches the cancer cell 201, 202, 204, 211.

The exosomal transport of doxorubicin may be used primarily to treat patients diagnosed with breast cancer 202, 203, 212. This form of drug administration turns out to be of particular importance in human epidermal growth factor receptor 2 (HER2) positive tumours which, compared to HER2 negative cells, preferentially take up exosomes with doxorubicin 203, 212. Moreover, doxorubicin-carrying exosomes can be potentially used in CRC 204 and the central nervous system neoplasm 205. This was demonstrated by Yang T. *et al.*, who proved that exosomes with doxorubicin and paclitaxel can cross the blood-brain barrier (BBB) in a zebrafish (danio rerio) model 205.

Exosomes can also become a platform for paclitaxel transport to neoplastic cells, again especially in breast cancer patients 206, 207. This form of exosomes shows strong anti-tumour properties *in vivo* 206, 207 even in the case of distant neoplastic metastases, although the level of paclitaxel in exosomes was about 1000 times lower than that in the case of standard drug administration 207. Paclitaxel-containing exosomes may also show anti-tumour efficacy in glioblastoma 208 and pulmonary metastases 209.

Finally, Liang G. *et al*. showed that exosomes expressing miR-21 inhibitor oligonucleotide (miR-21i), additionally enriched with 5-FU, exhibit anti-tumour properties, which are manifested by inhibition of the cell cycle and cell proliferation, with the simultaneous intensification of apoptosis 210. This observation may be of clinical relevance in the treatment of CRC patients 210. Furthermore, exosomes to which gemcitabine has been attached in treating pancreatic cancer patients may find clinical application 211.

## EVs - the dark side

Many interesting findings had been reported on EVs, but being scientist means to be critical too and to address questions. One of the major questions may be if a similar high degree of standardization is achieved on isolation and purification of EVs as in case of the FDA approved CellSeach^TM^ 48? The answer is that EV isolation and purification is steadily evolving and many various methods are in use as reviewed in depth by others 213-215. Typically, EVs may be isolated by various methodologies depending on their natural properties as size and affinity 216. Five of those were recently evaluated and their clinical applicability compared 217. The latest MISEV guidelines as published in *Journal of Extracellular Vesicles* (JEV) by the International Society For Extracellular Vesicles (ISEV) which is a professional society of researchers and scientists of EVs, is giving guidance on how to achieve a high degree of purity and standardization and reproducibility of EVs 77. However, some research indicates that EV content on protein level may be modulated depending on chosen EV purification method, though EV donor cells had been the same 217, 218. It appears that, for example, precipitation or ultracentrifugation of EVs could result in so-called 'touched EVs' that are either wrapped in polyethylene glycol (PEG, a polymer-based EV purification method) or potentially damaged by physical assault with high g-forces of 100k 219, 220. Lately a new gold standard might be achieved since steadily more and more researchers are taking advantage of somewhat 'untouched EVs' that were low g isolated followed by size exclusion chromatography (SEC) likely avoiding physical damage or aggregation of those EVs 214, 221, 222. Overall the EV field is still diverse and fluent on standardization, likely affecting reproducibility of earlier published historical EV data, that might be very well differing from newest EV data on the same topic while now MISEV guidelines were followed or SEC applied. Nevertheless, this is somewhat expected since EV research was 2 decades before a research field reserved and occupied by EV 'nerds'. For example, 2008 approximately 210 publication on exosome(s) were deposed on PubMed, 2021 approximately 5073 (search term exosomes). A similar tendency and total numbers are observable in case of large EVs (search term microvesicles).

On the contrary, CTCs might be a more developed concept and CTC research is highly benefiting due to the fact that CTCs has an historical advantage due to FDA's approval as granted 2004 as discussed above 48. Thus, Hillig T. *et al.* compered CTC numbers as accounted by two different methodologies and published in fact that CytoTrack^TM^ and CellSearch^TM^ reviled similar number of CTCs, and similar abilities to identify CTC *in vitro* 223. If EVs will ever reach such robustness permitting cross comparism on exact numbers remains open. It might be highly speculative, but the first FDA approval of EVs as a biomarker in screening and diagnosis might bring the needed guidance on standardization in spite of all efforts by ISEV and its members. As it stands for now, FDA issued a public safety notification on exosome products on December the 6^th^, 2019 (https://www.fda.gov/vaccines-blood-biologics/safety-availability-biologics/public-safety-notification-exosome-products).

## Conclusions

Liquid biopsies do promise a lot and are surely the future as seen during the ongoing SARS-CoV2 pandemic, in which a liquid biopsy made a huge impact on maintaining public health. So, what kind of arrows do we possess in our arrow quiver and which of those might be the sharpest to contest cancer? Assuming that we hit the cancer.

In case of liquid biopsies in cancer we have several, two of them, probably with the biggest perspective and future for a successful application associated with a robust high sensitivity and specificity might be CTCs and EVs. The first one already FDA approved for certain kind of cancer entities, predominantly from epithelial cancer entities with a metastatic nature and the latter one, EVs, that is highly experimental but something new, surely not fully developed and hence associated with big promises.

Both liquid biopsies are contested by its adversary, a highly capable individual beast, the whole cancer with its disconnected CTCs or cancer cells that are shedding EVs constantly for several purposes, mainly to ensure cancer survival and propagation within the host's body. Somehow a one-way direction with an unpleasant ending.

However, we have the tools to make a change. Two of them, CTCs and EVs as discussed in depth and compared. Interestingly, both, CTCs and EVs can be seen as a tool that cancer is using against us, but at the same time it's a tracer for cancer screening, cancer diagnosis, therapy monitoring sorting responders from non-responders as depicted in figure 1. Additionally, individual capabilities of CTCs and EVs are given, that are not shared between both. CTCs may be expended in various means as tumouroids for testing drug sensitivity helping us to tackle cancer. In case of EVs probably their usage as a vector for cancer therapies delivery which may be a promising vision that needs a lot of research to be done. Surely, EVs are a multiplier granting us cancer screening.

Cancer evolution uses both EVs and CTCs for cancer's survival and gaining advantage. We should take advantage of CTCs and EVs too! We should not consider EVs and CTCs just simple biomarkers as discussed above. Both EVs and CTCs orchestrate important processes of carcinogenesis. It seems that EVs are more profound during initiating process that are linked to carcinogenesis while CTCs later on. But, both have the same goal: cancer evolution. We might break cancer's momentum and take advantage. Depending on the tumour stage CTs and EVs could take a prominent future role as a liquid biopsy that fulfill its promise. Hence, the question “Who is better: EVs or CTCs?”, isn't of importance. The one that will help each cancer patient to become tumour free, free of secondary tumour loci is the true winner as a highly individual personalized medicine that includes as a first step cancer screening of note. Arnold Schwarzenegger said once in one of his movies: “If it can bleed, we can kill it.”(McTiernan J. (Director). (1987). PREDATOR [Film]. 20th Century Fox.). We might say once, “if it can release CTCs and EVs, we can heal it!”
